# Use of NSAIDs in orthopedic pain management: a Delphi consensus promoted by the Italian Society of Orthopedics and Traumatology (SIOT)

**DOI:** 10.3389/fpain.2026.1944515

**Published:** 2026-10-16

**Authors:** Paolo Tranquilli Leali, Alberto Di Martino, Michele Mercurio, Maria Silvia Spinelli, Flaminia Coluzzi, Francesca Gargano, Lisa Berti, Fabrizio Rivera, Paolo Grossi, Giuseppe Solarino, Alessandro Urbani, Francesco Scaglione, Pietro Simone Randelli

**Affiliations:** 1 FBSE, University of Sassari, Sassari, Italy; 2 Department of Biomedical and Neuromotor Sciences (DIBINEM), University of Bologna, Italy; 3 1st Orthopedic Department, IRCCS-Rizzoli Orthopedic Institute, Bologna, Italy; 4 Department of Orthopaedic and Trauma Surgery, Magna Græcia University, Renato Dulbecco University Hospital, Catanzaro, Italy; 5 Research Center on Musculoskeletal Health (MusculoSkeletalHealth@UMG), Magna Græcia University, Catanzaro, Italy; 6 Orthopaedic Oncology Unit, ASST Gaetano Pini-CTO, Milan, Italy; 7 Department of Surgical and Medical Sciences and Translational Medicine, Sapienza University of Rome, Rome, Italy; 8 Department of Life Sciences, Health and Health Professions, Link Campus University, Rome, Italy; 9 Department of Anesthesiology and Intensive Care, Campus Bio-Medico Foundation Rome, Italy; 10 Cottolengo Hospital, Turin, Italy; 11 Department of Anaesthesia and Intensive Care, ASST Gaetano Pini-CTO, Milan, Italy; 12 University of Bari, Bari, Italy; 13 Freelance Instructor and Facilitator, Venice, Italy; 14 University of Milan, Milan, Italy

**Keywords:** ketoprofen lysine salt, modified Delphi methodology, multimodal analgesia, NSAID prescribing appropriateness, perioperative pain management

## Abstract

**Introduction:**

Non-steroidal anti-inflammatory drugs (NSAIDs) are a cornerstone of pain management in orthopedics, yet significant variability persists in prescribing practices and adherence to guidelines.

**Methods:**

A two-round modified Delphi consensus was conducted under the coordination of a methodologist, involving a scientific board of seven members and a broader expert panel of orthopedic surgeons, anesthesiologists, and pharmacologists affiliated with the Italian Society of Orthopedics and Traumatology (SIOT). Twenty statements spanning four domains-NSAID mechanisms of action, perioperative pain management in elective surgery, traumatic pain, and non-oncological/oncological pain-were submitted for evaluation using a six-point Likert scale; consensus was defined as ≥75% of ratings concentrating within a single tertile.

**Results:**

Of the 20 statements, 12 reached consensus in the first round; six revised statements reached consensus in the second round; and two were eliminated because of persistent opinion dispersion. Final recommendations endorse short-cycle NSAID therapy (7–10 days) within multimodal analgesia, support the use of symptomatic slow-acting drugs for osteoarthritis to reduce NSAID consumption in osteoarthritis, recommend NSAIDs perioperatively in frail fracture patients to limit opioid use and delirium, identify ketoprofen lysine salt as having a favorable risk-benefit profile for postdischarge pain, and emphasize individualized NSAID selection based on cardiovascular, renal, and antiplatelet risk factors.

**Discussion:**

This SIOT consensus provides evidence-informed, clinically actionable recommendations for NSAID use across the principal orthopedic pain scenarios, aiming to improve prescribing appropriateness and patient safety in routine practice.

## 1 Introduction

Acute and chronic pain syndromes rank among the most common clinical presentations in orthopedic medicine, representing the predominant driver of healthcare-seeking behavior (1).

In particular, osteoarthritis (OA) is among the most prevalent musculoskeletal disorders worldwide and one of the leading causes of chronic pain and disability, imposing an increasing burden with far-reaching consequences at both the individual and societal level (2–4).

The pathophysiology of acute orthopedic pain involves nociceptive pathway activation secondary to tissue damage, typically in the context of traumatic injury and inflammatory responses. Peripheral sensitization is the key feature of acute pain, when inflammatory cytokines and pain-related molecules contribute to the activation of peripheral nerve terminals. However, if inadequately treated, acute pain may evolve into chronic pain, through mechanisms of peripheral and central sensitization, when the repetitive firing of primary afferent fibers triggers central amplification in the dorsal horn (5).

The increased spontaneous neuronal activity and the lowering threshold in the central nervous system (CNS), together with inadequate activation of the inhibitory descending pathway, contribute to pain chronification (6). In contrast, chronic pain is primarily caused by degenerative joint and musculoskeletal conditions, which represent a major clinical and socioeconomic burden.

Chronic musculoskeletal pain is mainly associated with central sensitization phenomena, characterized by an elevated responsiveness to normal afferent inputs and aberrant nociceptive signaling within the central nervous system. However, it should be noted that the inflammatory component is often responsible for pain flare-ups (1, 7).

Because OA is a complex multifactorial disease, different phenotypes of OA have been described, in order to identify patients who would benefit from specific treatments. In knee OA, indeed, six phenotypes have been identified: minimal joint disease, malaligned biomechanical (or mechanical overload), chronic pain, inflammatory, metabolic disorder, and bone and cartilage metabolism (8). Similarly, various pain phenotypes have been described, including nociceptive, neuropathic, and nociplastic pain in OA (9).

As such, pain intensity and quality, including signs of sensitization, may serve as reliable indicators of underlying disease severity and progression, offering utility in both prognostication and treatment monitoring (10). However, clinicians must recognize pain as an independent clinical condition warranting specific therapeutic interventions beyond treatment of the primary disease process. Indeed, as subjective and sensorial experience, pain varies greatly not only from person to person but also within the same subject. Genetics, prior learning, expectations, sociocultural environments, and psychological factors can influence the pain experience, and a multidisciplinary approach is necessary. In this context, multimodal analgesia, by targeting different pain pathways, is currently considered the standard of care for effective and opioid-sparing pain management. Pain also affects the quality of life and patients present an impaired global functioning, particularly relating to the physical, psychological, and social aspects.

Non-steroidal anti-inflammatory drugs (NSAIDs) are a primary therapeutic option for treating pain in orthopedics due to their effectiveness in reducing inflammation and alleviating pain associated with several musculoskeletal conditions (11–13). However, their use is often accompanied by safety concerns, particularly regarding gastrointestinal, cardiovascular, and renal adverse events (14–16). These risks are strongly influenced by dose, duration of therapy, and patient-specific factors, highlighting the importance of individualized treatment strategies. Moreover, there are significant differences in prescribing practices and adherence to existing guidelines, which may affect treatment appropriateness and effectiveness (17).

The Delphi method is a structured, iterative consensus-building technique originally developed at the RAND Corporation in the early 1950s, designed to elicit and aggregate expert opinion on topics for which robust empirical evidence is lacking or incomplete (18). The method relies on four core principles: anonymity of participants, iterative questioning across successive rounds, controlled feedback provided between rounds, and statistical aggregation of group responses. In each round, panelists independently rate or evaluate a series of statements; their aggregated responses are then shared with the group, allowing participants to revise their judgments in light of the collective feedback. This process continues until a predefined consensus threshold is reached or a set number of rounds is completed. In the health sciences, the Delphi method has gained widespread adoption for the development of clinical guidelines, recommendation statements, and research priorities, particularly in contexts where randomized controlled trial evidence is unavailable or difficult to obtain (19–21). This methodology was also applied to other studies on pain management (22).

The current study aimed to establish an expert consensus on key clinical questions regarding NSAID use in orthopedic pain management through a structured Delphi process under the coordination of a methodologist, involving experts from the Italian Society of Orthopedics and Traumatology (SIOT), pharmacologists, and anesthesiologists. The decision process in the production of the statements is reported and commented in the discussion. These recommendations would improve prescribing appropriateness, treatment effectiveness, and patient safety.

## 2 Methods

A consensus study was conducted utilizing a two-round modified online Delphi technique based on the classic Delphi methodology (Figure 1).

**Figure 1:**
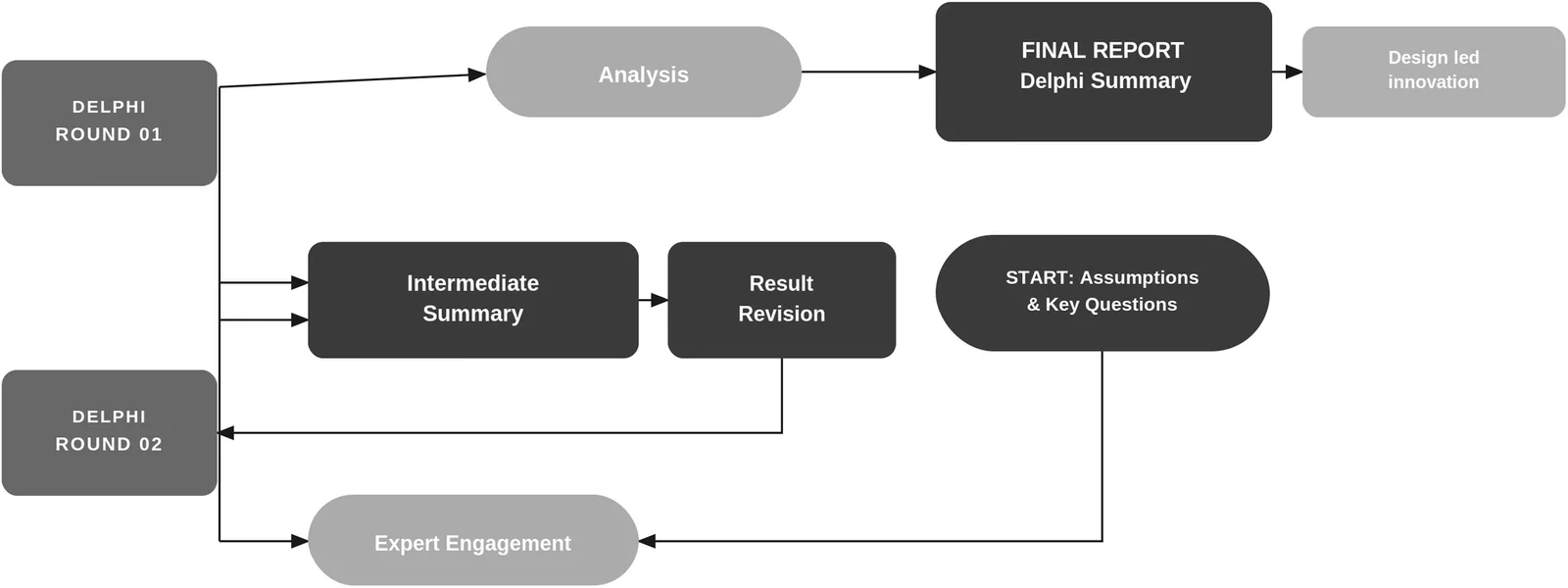
A schematic representation of consensus development.

The scientific board consisted of seven members: six clinicians (four orthopedic surgeons, one pharmacologist, and one anesthesiologist with established expertise in perioperative pain management) and one methodologist (Table 1). The scientific board was responsible for defining the clinical questions, identifying key topics, and conducting the literature search required to develop the Delphi questionnaire.

**Table 1:** Members of the board (B) and the panel (P).

Name	Occupation	Affiliation	Board and/or panel member
Alessandro Aprato	Orthopedic surgeon	Ospedale Regina Margherita di Torino	P
Andrea Audisio	Orthopedic surgeon	Ospedale Regina Margherita di Torino	P
Giorgia Lucia Benedetto	Biotechnologist	Università “Magna Graecia” di Catanzaro	P
Lisa Berti	Rehabilitation medicine	Istituto Ortopedico Rizzoli, Bologna	P
Romolo Borgese	Orthopedic surgeon	Azienda Ospedaliera Universitaria Integrata di Verona	P
Federica Bressi	Rehabilitation medicine	Fondazione Policlinico Universitario Campus Bio-Medico di Roma	P
Flaminia Coluzzi	Anesthesiologist	Università “La Sapienza” di Roma	P
Riccardo Compagnoni	Orthopedic surgeon	Università degli Studi di Milano	P
Fabio D’Angelo	Orthopedic surgeon	Università degli Studi dell’Insubria, Varese	P
Alberto Di Martino	Orthopedic surgeon	Istituto Ortopedico Rizzoli, Bologna	B, P
Paolo Ferrua	Orthopedic surgeon	Università degli Studi di Milano	P
Francesca Gargano	Anesthesiologist	Fondazione Policlinico Universitario Campus Bio-Medico di Roma	P
Giuseppe Geraci	Orthopedic surgeon	Istituto Ortopedico Rizzoli, Bologna	P
Stefano Giaretta	Orthopedic surgeon	Azienda ULSS 8 Berica, Regione del Veneto	P
Michele Grasso	Orthopedic surgeon	AISOT President	P
Paolo Grossi	Anesthesiologist	ASST G. Pini/CTO, Milano	B, P
Michele Mercurio	Orthopedic surgeon	Università “Magna Græcia”, Catanzaro	B, P
Lucrezia Moggio	Rehabilitation medicine	Ospedale di Biella-ASLBI	P
Pietro Simone Randelli	Orthopedic surgeon	Università degli Studi di Milano	P
Fabrizio Rivera	Orthopedic surgeon	Ospedale Cottolengo di Torino	P
Barbara Rossi	Researcher	ISS-Istituto superiore di Sanità	P
Carlo Ruosi	Orthopedic surgeon and rehabilitation medicine	Università degli Studi di Napoli	P
Elena Manuela Samalia	Orthopedic surgeon	Università degli Studi di Verona	P
Francesco Scaglione	Pharmacologist	Università degli Studi di Milano	B, P
Giuseppe Solarino	Orthopedic surgeon	Università degli Studi di Bari	P
Maria Silvia Spinelli	Orthopedic surgeon	ASST G. Pini/CTO, Milano	B, P
Niccolò Stefanini	Orthopedic surgeon	Istituto Ortopedico Rizzoli, Bologna	P
Paolo Tranquilli Leali	Orthopedic surgeon	^1^F-Medical Group, Frosinone	B, P
Alessandro Urbani	Methodologist	Freelance Instructor and Facilitator	B

A literature review was performed using PubMed/MEDLINE with the following search terms: “Anti-Inflammatory Agents, Non-Steroidal” [Mesh], “Orthopedics” [Mesh], “Orthopedic Procedures” [Mesh], “Musculoskeletal Diseases” [Mesh], “Pain Management” [Mesh], “Pain, Postoperative” [Mesh], “Musculoskeletal Pain” [Mesh], “Bone and Bones/drug effects” [Mesh], “Fracture Healing/drug effects” [Mesh], “Osteogenesis/drug effects” [Mesh], “Anti-Inflammatory Agents, Non-Steroidal/adverse effects” [Mesh]; NSAIDs, non-steroidal anti-inflammatory drugs, cyclooxygenase (COX)-2 inhibitors, symptomatic slow-acting drugs for osteoarthritis (SYSADOAs), orthopedic, orthopedic surgery, fracture, musculoskeletal pain, postfracture, trauma, sports medicine, joint pain, pain management, pain control, postoperative analgesia, perioperative, multimodal analgesia, rehabilitation, bone healing, delayed union, and pseudoarthrosis. The PubMed/MEDLINE search was limited to articles published within the last 15 years.

The scientific board evaluated the retrieved articles according to their level of evidence, selecting those with the highest evidence level for each topic to inform the initial statement development.

The Delphi voting process was conducted in two online sessions held on 29 October 2025 (first round) and 3 December 2025 (second round), with panel members all present in a virtual meeting. The voting panel comprised the six clinical members of the scientific board and 22 additional experts (16 orthopedic and trauma surgeons, two anesthesiologists, one biotechnologist, one neurologist, one acupuncturist physician, and one rehabilitation specialist). The list of group members who participated in the rounds of vote is given in Table 1. Participants expressed their level of agreement with each statement using a six-point Likert scale (23, 24). The methodologist analyzed and summarized the results, while keeping the individual evaluations anonymous.

Ratings obtained via Likert scale were stratified into three tertiles of ascending relevance (I–III): negative/low (rating 1–2), neutral/moderate (rating 3–4), and positive/high (rating 5–6). The agreement threshold was set at ≥75% of participants' ratings concentrating within a single tertile. The thresholds were set at the beginning of the study. Disagreement was identified when rating distribution across all tertiles reached ≥80% without achieving the agreement threshold. When substantial heterogeneity prevented attainment of these predefined cutoffs, indicating opinion dispersion, the statement was eliminated from the final recommendation set.

These metrics quantified the degree of consensus for each statement and informed decisions regarding statement revision or acceptance.

Upon completion of the first voting round, the board members reviewed the voting results and panel comments; statements achieving consensus were incorporated into the final recommendations. Statements failing to achieve agreement underwent collective board discussion and were modified where necessary to improve comprehension. These revised statements were subsequently presented for evaluation in the second round.

## 3 Results

### 3.1 Statement definition and the Delphi process

Based on the results of the systematic literature review and preliminary discussions, the scientific board proposed 20 statements (Supplementary Material), which were divided into four domains (Table 2), and answered four clinical questions (Table 3). This set of statements was internally validated by the scientific board before submission to the expert panel for voting.

**Table 2:** Questionnaire domains.

Domain number	Domain subject
Domain 1	NSAID mechanisms of action in pain management in orthopedics
Domain 2	Medical pain therapy in orthopedics for patients undergoing elective orthopedic surgery
Domain 3	Medical therapy of pain in the orthopedic patient with traumatic pain
Domain 4	NSAIDs in the management of oncological and non-oncological pain in orthopedics

**Table 3:** Clinical questions.

Question number	Question subject
Clinical question 1	What are the mechanisms of action of NSAIDs in pain control in orthopedics?
Clinical question 2	The patient's course in elective orthopedic surgery: Is it possible to define a virtuous algorithm for NSAIDs in orthopedics that accompanies patients with pain throughout all stages?
Clinical question 3	The patient's course in traumatology: Is it possible to define a virtuous algorithm for NSAIDs in orthopedics that accompanies the patient with a fracture through all stages?
Clinical question 4	Criteria for choosing NSAIDs: Which NSAID is most appropriate for chronic non-cancer pain? Which NSAID is most appropriate for chronic cancer pain?

Twenty-seven out of 28 panel members participated in the first Delphi round and 23/28 participated in the second round. In the first round, agreement was reached on 12 statements, which were therefore included in the final results. Six of the remaining eight statements were modified, taking into account the comments contained in the panel's responses, and examined in the second round: all of these statements reached consensus in their modified form. Conversely, two statements (six and eight, both pertaining to the second domain) were eliminated because of dispersion of opinions.

### 3.2 First round

In the *first domain* (NSAID mechanisms of action in pain management in orthopedics), agreement was reached on 2/4 statements (Table 4, Supplementary Material). In particular, the scientific panel agreed on Statement 1 (NSAIDs act on the genesis of inflammatory pain, on spinal transmission, and activate descending inhibitory pathways, with class-specific effects) and 4 (the duration of NSAID therapy for the treatment of orthopedic pain should be limited in time, with cycles of a maximum of 7–10 days), and the treatment should be included within a multimodal approach.

**Table 4:** List of statements reaching agreement of the panel.

Domain number	Statement
Domain 1	NSAIDs act on the genesis of inflammatory pain, on spinal transmission, and activate descending inhibitory pathways, with class-specific effects
	The efficacy of NSAIDs in treating musculoskeletal and joint pain is similar when equivalent doses are used. The equivalent dose is the amount of drug that produces a similar analgesic and anti-inflammatory effect compared with another NSAID
	The gastrointestinal adverse events of systemic NSAIDs are dose-dependent and drug-specific for every route of administration except the topical one
	The duration of NSAID therapy for the treatment of orthopedic pain should be limited in time (cycles of a maximum of 7–10 days), and the treatment should be included within a multimodal approach
Domain 2	NSAID treatment for osteoarticular pain while awaiting surgery should be performed starting with the minimum effective doses and/or in repeated short cycles
	SYSADOAs, when administered as part of conservative treatment strategies for osteoarthritic pain, can help reduce the use of NSAIDs
	In patients with severe osteoarthritis (Kellgren–Lawrence >3) awaiting surgery, therapy may include treatment with NSAIDs or paracetamol combined with instrumental and manual physiotherapy techniques. Referral to a pain therapist may be appropriate
	Early functional recovery requires analgesic continuity with dose adjustment and, if needed, the addition of “rescue” medications, including NSAIDs, considering ERAS protocols
	At discharge after surgery, in the management of pain, ketoprofen lysine salt represents the NSAID with the best risk–benefit ratio especially in treatments lasting up to 7–14 days
	In patients with severe osteoarthritis, unresponsive to physiotherapy treatments, and intolerant to NSAIDs, the use of paracetamol and opioids is advisable, even in combination
Domain 3	In patients at risk of developing stress fractures, regular use of NSAIDs should be limited
	The use of NSAIDs in the perioperative period for frail patients undergoing surgery for fractures is recommended within multimodal analgesia protocols to reduce opioid intake and limit the onset of complications, including delirium
	Short-term administration (<10–15 days) of an NSAID in the postoperative period and upon discharge of a patient operated on for a fracture is not associated with a significant risk of pseudoarthrosis
Domain 4	The presence of cardiovascular risk factors or pathology in patients with chronic orthopedic pain requires a carefully personalized choice of NSAID
	Ketoprofen has a lower cardiovascular risk than other NSAIDs
	The potential interference of NSAIDs with platelet aggregation processes requires particular attention in patients receiving ASA therapy
	The presence of impaired renal function in patients with chronic orthopedic pain requires a carefully personalized choice of NSAID, and the patient's hydration must be monitored
	The control of oncological pain in orthopedics may include the use of NSAIDs as part of a multimodal analgesic strategy

In the *second domain* (medical pain therapy in orthopedics for patients undergoing elective orthopedic surgery), agreement was reached on four of eight statements (Table 4, Supplementary Material). In particular, the scientific panel agreed on Statement 5 (NSAID treatment for osteoarticular pain while awaiting surgery should be performed starting with the minimum effective doses and/or in repeated short cycles), 9 (in patients with severe osteoarthritis—Kellgren–Lawrence >3—awaiting surgery, therapy may include treatment with NSAIDs or paracetamol combined with instrumental and manual physiotherapy techniques. Referral to a pain therapist may be appropriate), 10 [early functional recovery requires analgesic continuity with dose adjustment and, if needed, the addition of “rescue” medications, including NSAIDs, considering enhanced recovery after surgery (ERAS) protocols], and 12 (in patients with severe osteoarthritis, unresponsive to physiotherapy treatments, and intolerant to NSAIDs, the use of paracetamol and opioids is advisable, even in combination).

In the *third domain* (medical therapy of pain in the orthopedic patient with traumatic pain), agreement was reached on 2/3 statements (Table 4, Supplementary Material). In particular, the scientific panel agreed on Statement 14 (the use of NSAIDs in the perioperative period for frail patients undergoing surgery for fractures is recommended within multimodal analgesia protocols to reduce opioid intake and limit the onset of complications, including delirium) and 15 [short-term administration (<10–15 days) of an NSAID in the postoperative period and upon discharge of a patient operated on for a fracture is not associated with a significant risk of pseudoarthrosis].

In the *fourth domain* (NSAIDs in the management of oncological and non-oncological pain in orthopedics), agreement was reached on 4/5 statements (Table 4, Supplementary Material). In particular, the scientific panel agreed on Statement 16 (the presence of cardiovascular risk factors or pathology in patients with chronic orthopedic pain requires a carefully personalized choice of the NSAID), 18 [The potential interference of NSAIDs with platelet aggregation processes requires particular attention in patients receiving acetylsalicylic acid (ASA) therapy], 19 (the presence of impaired renal function in patients with chronic orthopedic pain requires a carefully personalized choice of NSAID and the patient's hydration must be monitored), and 20 (the control of oncological pain in orthopedics may include the use of NSAIDs as part of a multimodal analgesic strategy).

### 3.3 Second round

In the second round, the modified versions of the six statements on which agreement had not previously been reached were all approved by the panel of experts (Table 4, Supplementary Material). Specifically, in the *first domain*, agreement was reached on modified statements 2 (the efficacy of NSAIDs in treating musculoskeletal and joint pain is similar when equivalent doses are used. The equivalent dose is the amount of drug that produces a similar analgesic and anti-inflammatory effect compared with another NSAID) and 3 (the gastrointestinal adverse events of systemic NSAIDs are dose-dependent and drug-specific for every route of administration except the topical one). In the *second domain*, agreement was reached on modified statements 7 (SYSADOAs, when administered as part of conservative treatment strategies for osteoarthritic pain, can help reduce the use of NSAIDs) and 11 (at discharge after surgery, in the management of pain, ketoprofen lysine salt represents the NSAID with the best risk–benefit ratio especially in treatments lasting up to 7–14 days). In the *third domain*, agreement was reached on modified statement 13 (in patients at risk of developing stress fractures, regular use of NSAIDs should be limited). In the *fourth domain*, agreement was reached on modified statement 17 (ketoprofen has a lower cardiovascular risk than other NSAIDs).

## 4 Discussion

This study outlines the results of an expert consensus meeting that reported the recommendations for short-term NSAIDs therapy of pain in adult orthopedic patients.

This study is subject to the well-recognized limitations of the Delphi method (25), including its limited sensitivity and replicability—outcomes being highly dependent on panelists' opinions and panel size—as well as the selection biases inherent to the purposive sampling of experts (26). In particular, the exclusion of some statements after the first round of voting—resulting from failure to achieve consensus and considerable divergence of expert opinion—may represent a further limitation of the present study.

In the *first domain*, related to NSAID mechanisms of action in pain management, a consensus was reached on the fact that NSAIDs demonstrate class-specific effects, including modulation of inflammatory pain generation, spinal nociceptive transmission, and activation of descending inhibitory systems (*Statement 1*). The mechanisms of action of NSAIDs have been the subject of several reviews (11, 27). NSAIDs provide analgesia by inhibiting the COX family of enzymes, which are involved in the formation of prostaglandins in the periphery and CNS (27). Beyond cyclooxygenase inhibition, NSAIDs may also modulate additional inflammatory pathways and nociceptive processing, contributing to their overall analgesic effect. The relative contribution of peripheral and central effects to overall analgesia depends on the site of administration and the biochemical properties (e.g., the ability to cross the blood–brain barrier) of specific NSAIDs (28).

Prostaglandins, particularly prostaglandin E2 (PGE2), promote the inflammatory response in the periphery and the sensitized neuronal state in the dorsal horn of the spinal cord (11). The activation of PGE2 receptors [EP2; G protein-coupled receptors that modulate the production of cyclic adenosine monophosphate (cAMP) and the activity of protein kinases A and C] causes a change in the conductance of several ion channels, including sodium channels, voltage-gated potassium channels, and transient receptor potential channels (29). At peripheral sites of inflammation, the activation of PGE2 receptors on nociceptor terminals causes neuronal depolarization and sensitization, facilitating their activation by other inflammatory mediators such as bradykinin (30). In response to nociceptive input, COX-2 expression is upregulated in several areas of the CNS, and PGE2 is released in the dorsal horn, considered a major site for PGE2 action (31). PGE2 is thought to have both pre- and postsynaptic effects that contribute to the central sensitization of neurons in the dorsal horn. PGE2 acts on nociceptor presynaptic nerve terminals to increase the opening of voltage-gated calcium channels, thereby promoting the release of pronociceptive neurotransmitters (e.g., glutamate, substance P, calcitonin gene-related peptide, and brain-derived neurotrophic factor) from nociceptors; it also acts postsynaptically to enhance the depolarization of second-order neurons (27). PGE2 can also act on EP2-like receptors in the nerve terminals of inhibitory interneurons in the dorsal horn to reduce glycine release. Given the constitutive role of glycine in regulating the excitability of dorsal horn neurons, this would predictably lead to a state of increased excitability and facilitation of pain signaling (32). Finally, there is some evidence that NSAIDs act centrally in structures such as the periaqueductal gray and the rostral ventromedial medulla to activate descending inhibitory pain pathways (33).

The *second statement* was refined in the second round to affirm that NSAIDs demonstrate similar efficacy in treating musculoskeletal and joint pain when equivalent doses are administered. The comparative effectiveness of NSAIDs has been indicated in a document edited by the Agency for Healthcare Research and Quality (34). Similarly, to standardize the quantification of NSAID consumption in arthritis research, ASAS (Assessment of SpondyloArthritis International Society) designed a specific NSAID intake score for use in clinical trials and epidemiological studies (35).

The *third statement* was also refined, establishing that the gastrointestinal adverse events of systemic NSAIDs are dose-dependent and drug-specific for every route of administration, except the topical one. In fact, the effect of systemic NSAIDs on the gastrointestinal tract depends on their mechanism of action, and risk factors for NSAID gastrointestinal complications have been identified (36). COX-2 inhibitors are generally considered safer than non-selective NSAIDs. However, a review of 7,616 patients showed that although COX-2 inhibitors significantly reduced perforation, obstruction, and bleeding risk vs. ns-NSAIDs (non-selective NSAIDs) plus proton pump inhibitors (RR, 0.38; 95% CI, 0.25–0.56; *P* < 0.001), this advantage was confined to high-risk patients and long-term users (37). The gastrointestinal safety of topical NSAID formulations has been reviewed in several studies and meta-analyses (38).

The *fourth statement* reached consensus in the first round, establishing that NSAID therapy should be restricted to short cycles of 7–10 days and incorporated into a multimodal treatment strategy. In fact, because renal, cardiovascular, and gastrointestinal risks are closely related to NSAID dose and duration of treatment, current recommendations suggest that NSAIDs may be considered relatively safe when prescribed at the lowest effective dose for a period not exceeding 10 days (39–43). Multimodal analgesia combinations incorporating NSAIDs have been established as an effective pain management strategy in several studies, particularly in the perioperative setting (44, 45).

Within the *second domain*, which addressed pain management in patients awaiting elective orthopedic surgery, the *statement 5* achieved consensus in the first round, advocating for the initiation of NSAID therapy at the minimum effective dose and/or in repeated short cycles for the treatment of osteoarticular pain before surgery (20, 39–43, 46). NSAIDs can be used for managing recurrent acute OA flares, commonly described as the typical natural history of symptomatic OA progression, which can be distressing and disabling. These flares should be viewed and managed as an “acute-on-chronic” condition (47).

The *sixth statement*, concerning the potential benefits of SYSADOA use during intervals between therapy cycles or in patients with NSAID contraindications, was eliminated after the first round because of a lack of consensus and divergent opinions.

The *seventh statement* was refined in the second round to establish that SYSADOAs can reduce NSAID consumption when integrated into conservative treatment strategies for osteoarthritic pain. A recent meta-analysis confirmed the efficacy of SYSADOAs in osteoarthritis (48), with evidence supporting their NSAID-sparing effect in this clinical context (49–51).

The *eighth statement*, concerning the use of multimodal therapy with NSAIDs or paracetamol combined with intra-articular injection techniques using hyaluronic acid or PRP in patients with moderate osteoarthritis awaiting surgical intervention, was eliminated after the first round because of a lack of consensus and divergent opinions. Notably, a recent SIOT position statement endorses intra-articular hyaluronic acid for chronic knee OA in the absence of acute flare and PRP for symptomatic knee OA when administered at highly specialized centers (52).

*Statement 9* reached consensus in the first round, recommending that patients with severe osteoarthritis awaiting surgery may receive combined therapy consisting of NSAIDs or paracetamol plus instrumental and manual physiotherapy techniques. The use of NSAIDs or paracetamol in severe osteoarthritis has been recommended in several guidelines (43, 53). The possible combination of drug therapy with instrumental and manual physiotherapy techniques has been suggested (54–56). Referral to a pain therapist may be appropriate in this setting (57, 58).

Consensus was similarly achieved in the first round for *statement 10*, which established that early functional recovery necessitates analgesic continuity with appropriate dose titration and, where indicated, supplementation with rescue medications such as NSAIDs, in line with ERAS protocols. The appropriate use of analgesics to promote early functional recovery after orthopedic surgery has been outlined (59–61). The use of opioid analgesics should be minimized, while NSAIDs can be beneficial for pain control while reducing opioid-related side effects.

*Statement 11* was refined in the second round to identify ketoprofen lysine salt as the NSAID with the most favorable risk-benefit profile among NSAIDs for postdischarge surgical pain management, especially for treatments lasting 7–14 days. The efficacy of ketoprofen for acute postoperative pain has been supported by a Cochrane review (62). In fact, ketoprofen lysine salt has emerged as a NSAID with notable efficacy and safety advantages. Evidence from multiple clinical trials demonstrates that ketoprofen achieves the highest ratio between anti-inflammatory and analgesic effects, conferring superior potency compared with other non-selective NSAIDs in relieving moderate to severe pain, improving functional status, and enhancing overall patient condition (21, 63, 64). A recent review outlined the advantages of the use of ketoprofen lysine salt vs. ketoprofen acid in terms of greater gastric tolerability and quicker onset of action (65). Finally, it is known that NSAIDs increase cardiovascular risk. Ketoprofen has proven to be one of the most tolerable NSAIDs for cardiovascular disease. In particular, a large meta-analysis has shown that ketoprofen (OR: 0.86–1.16) carries a lower cardiovascular risk than other NSAIDs such as etoricoxib (OR: 1.24–1.57), diclofenac (1.22–1.34), and ketorolac (OR: 1.49–2.18) (66, 67).

The *12th statement* reached consensus in the first round, establishing that paracetamol and opioid therapy, either as monotherapy or in combination, is advisable for patients with severe osteoarthritis who demonstrate inadequate response to physiotherapy and intolerance to NSAIDs. This recommendation was derived from multiple clinical guidelines (13, 43, 53, 68). Notably, in statement 8, the recommendation on the use of intra-articular hyaluronic acid—which had been proposed alongside NSAIDs for knee OA in a recent publication (69)—was withdrawn after the first round of voting, owing to a lack of consensus and divergent expert opinion.

Within the *third domain*, which addressed medical management of traumatic pain in the orthopedic patient population, *statement 13* was refined in the second round to establish that regular use of NSAIDs should be limited in patients at risk of developing stress fractures. In fact, there is emerging evidence suggesting that NSAIDs may inhibit remodeling within cortical bone which, in turn, could lead to increased bone density with extended use in a similar fashion as antiresorptive medications; this mechanism could increase the risk of stress fractures in physically active populations (70, 71).

The *14th statement* reached consensus in the first round, establishing that NSAIDs are recommended in the perioperative period for frail patients undergoing fracture surgery as part of multimodal analgesia protocols to reduce opioid consumption and limit complications, including delirium. In a recent RCT trial, the combination of oral acetaminophen and intravenous ketorolac was a viable alternative to opioid-based pain management in closed tibial shaft fractures treated with intramedullary nailing, decreasing opioid consumption without affecting pain scores or union rates (72). In another RCT trial, conducted in 132 patients aged ≥65 years undergoing hip fracture surgery, the prevalence of postoperative delirium was significantly lower in the anti-inflammatory bundle group (containing NSAIDs and glucocorticoids) compared with the placebo group (73).

*Statement 15* gained first-round consensus, affirming that brief postoperative NSAID treatment (<10–15 days) following fracture surgery and at discharge does not confer significant pseudoarthrosis risk. Several studies and meta-analyses have shown that short-term NSAID use does not significantly affect bone healing rates or increase the incidence of delayed union (12, 74, 75). However, experimental evidence suggests that prostaglandins play a role in bone remodeling and repair, providing a biological rationale for concerns regarding prolonged NSAID use (76).

In the *fourth domain*, which pertained to selection criteria for NSAID therapy in the management of oncological and non-oncological pain in orthopedics, *statement 16* achieved first-round consensus, establishing that cardiovascular risk factors or pathology in patients with chronic orthopedic pain require careful, personalized NSAID selection. The safety of NSAIDs with regard to cardiovascular events has been studied in a large number of clinical studies and meta-analyses, showing that NSAID use is associated with an increased risk of cardiovascular thrombotic events (including myocardial infarction), heart failure, and hypertension (67, 77–79). However, a recent systematic review questioned this assumption for hip and knee osteoarthritis, suggesting that FDA should reconsider the black-box warning on these agents (80).

*Statement 17* was refined in the second round to establish that ketoprofen has a lower cardiovascular risk than other NSAIDs. Several systematic reviews and meta-analyses suggest that ketoprofen has the lowest incidence of cardiovascular adverse events among NSAIDs (16, 67, 79).

Two meta-analyses of randomized trials have reported that both selective and non-selective COX-2 inhibitors are associated with an increased risk of serious ischemic vascular events, predominantly myocardial infarction (81, 82). Myocardial insufficiency has been associated with etoricoxib, lumiracoxib, and rofecoxib, as well as diclofenac and ibuprofen (77, 83), but not with naproxen (84). These findings suggest that mechanisms other than COX-2/COX-1 selectivity may be involved in cardiovascular toxicity. A large epidemiological study derived from a database of 8.5 million new NSAID users identified 79,553 cases of acute myocardial infarction. Among the NSAIDs with the highest risk, in addition to coxibs (rofecoxib, etoricoxib), the study found ketorolac and diclofenac, while ketoprofen appeared to be the most widely used drug with the lowest cardiovascular risk (67). A recent study shows that the mechanism of cardiotoxicity is likely non-COX-mediated. Interestingly, in this study, diclofenac, but not ketoprofen, appeared toxic to cardiac cells, despite the two drugs having comparable COX-2/COX-1 activity. Although both drugs increased ROS production, only diclofenac exposure decreased mitochondrial membrane potential, which, in turn, reduced the DC of the 26S proteasome, leading to cell death (85).

*Statement 18* gained first-round consensus, highlighting that potential NSAID-mediated interference with platelet aggregation requires special consideration in patients receiving antiplatelet therapy with aspirin (ASA). The concomitant use of low-dose aspirin for cardioprotection and NSAIDs for pain relief is quite frequent, especially in the elderly. Aspirin induces irreversible platelet inhibition through covalent acetylation of COX-1, while other NSAIDs produce reversible antiplatelet effects; furthermore, from a pharmacokinetic perspective, NSAIDs with long half-lives can compete with aspirin (which has a very short half-life) for COX-1 binding, potentially reducing its antiplatelet efficacy (86–88). Although direct competitive aspirin/NSAID interaction has been well documented and docking models based on this mechanism yield realistic results, the actual interaction mechanisms may be more complex (89, 90). *In vitro* studies using preincubation of platelet suspensions with NSAIDs followed by aspirin addition and agonist-induced platelet stimulation (e.g., with arachidonic acid) demonstrated that most NSAIDs—including naproxen, ibuprofen, flufenamate, and even COX-2 inhibitors such as celecoxib—reduced the antiplatelet effect of aspirin, whereas ketoprofen and diclofenac did not interfere with the activity of aspirin (89–92). The proposed hypothesis is that different NSAIDs afford differential impedance of the access of aspirin to the acetylation site (serine residue at position 529) in the platelet cyclooxygenase-1. Various strategies to mitigate the interfering effects of NSAIDs on aspirin-induced platelet aggregation inhibition have been advocated, including optimized dosing schedules and judicious NSAID selection (87, 90). However, the use of non-selective NSAIDs, particularly ibuprofen, in patients taking low-dose aspirin may impair its antithrombotic activity and cardioprotective effect, leading to a significant clinical risk, especially for those using aspirin for secondary prevention.

The *19th statement* reached consensus in the first round, establishing that the presence of renal impairment in patients with chronic orthopedic pain requires careful individualization of NSAID therapy and systematic monitoring of hydration status. The literature on NSAID renal effects has identified multiple risk factors for acute and chronic kidney injury (93–95). Nephrotoxicity risk is elevated in elderly patients, hypovolemia, high-dose NSAID exposure, concurrent use of vasoconstrictors (such as calcineurin inhibitors), and concomitant therapy with renin–angiotensin–aldosterone system inhibitors or diuretics. Selective COX-2 inhibitors also carry renal risks; therefore, clinicians should recommend short-term rather than prolonged NSAID therapy to minimize morbidity and mortality. These considerations further support a precision medicine approach in NSAID prescribing, balancing efficacy and safety according to individual patient risk profiles.

*Statement 20* gained first-round consensus, recommending that the management of oncological pain in orthopedic settings may incorporate NSAIDs as a component of multimodal analgesic therapy. The use of NSAIDs as a part of the analgesic treatment of pain in cancer patients has been outlined in several reviews and guidelines, even though high-quality studies are scarce (96–98). Emerging evidence has raised the possibility that NSAIDs may exert antitumor effects and improve postoperative outcomes, although prospective trials are needed to evaluate this hypothesis (99).

## 5 Conclusion

This consensus document represents the outcome of a Delphi project of the SIOT to establish consensus-based recommendations for NSAID utilization in the management of pain in the orthopedic patient. The engagement of specialists representing diverse Italian clinical hospitals facilitated the synthesis of complementary perspectives and heterogeneous clinical experiences. The recommendations proposed through this document, founded upon scientific evidence and informed by clinical practice, constitute a resource for delivering pragmatic guidance readily translatable into routine clinical practice.

## Data Availability

The original contributions presented in the study are included in the article/Supplementary Material, further inquiries can be directed to the corresponding author.
