# Supplementary material for: Use of NSAIDs in orthopedic pain management: a Delphi consensus promoted by the Italian Society of Orthopedics and Traumatology (SIOT)
Source: Front Pain Res (Lausanne). 2026 Oct 16;7:1944515. doi: 10.3389/fpain.2026.1944515 (PMC13720948; doi:10.3389/fpain.2026.1944515)
Supplement: Supplementary file 1 [file Table1.docx]

**Supplementary material**

**List of the statements submitted to the expert panel in the first round**

|  |  | Statement |
| --- | --- | --- |
| Domain 1 | **1** | NSAIDs act on the genesis of inflammatory pain, on spinal transmission, and activate descending inhibitory pathways, with class-specific effects |
|  | **2** | The efficacy of NSAIDs in musculoskeletal and joint pain is comparable at equivalent doses |
|  | **3** | Gastrointestinal adverse events of NSAIDs are dose-dependent and drug-specific, regardless of the route of administration |
|  | **4** | The duration of NSAID therapy for the treatment of orthopedic pain should be limited in time (cycles of a maximum of 7–10 days), and the treatment should be included within a multimodal approach |
| Domain 2 | **5** | NSAID treatment for osteoarticular pain while awaiting surgery should be performed starting with the minimum effective doses and/or in repeated short cycles. |
|  | **6** | During intervals between therapy cycles, or in patients with contraindications to the use of NSAIDs, the use of SYSADOA (Symptomatic Slow-Acting Drugs for Osteoarthritis) may be beneficial |
|  | **7** | SYSADOA (Symptomatic Slow-Acting Drugs for Osteoarthritis) can be used in combination with NSAIDs as part of conservative pain-management strategies. |
|  | **8** | In patients with moderate osteoarthritis (Kellgren–Lawrence up to grade 3) awaiting surgical intervention, multimodal therapy may include treatment with NSAIDs or paracetamol combined with intra-articular injection techniques using hyaluronic acid or PRP, provided they are performed at least three months before surgery |
|  | **9** | In patients with severe osteoarthritis (Kellgren–Lawrence >3) awaiting surgery, therapy may include treatment with NSAIDs or paracetamol combined with instrumental and manual physiotherapy techniques. Referral to a pain therapist may be appropriate. |
|  | **10** | Early functional recovery requires analgesic continuity with dose adjustment and, if needed, the addition of ‘rescue’ medications, including NSAIDs, considering ERAS protocols |
|  | **11** | In the context of social reintegration, in cases of acute pain with NRS >4, the most suitable NSAID to use as needed is ketoprofen lysine salt, in terms of risk–benefit ratio |
|  | **12** | In patients with severe osteoarthritis, unresponsive to physiotherapy treatments, intolerant to NSAIDs, the use of paracetamol and opioids is advisable, even in combination. |
| Domain 3 | **13** | In patients at risk of developing stress fractures, chronic use of NSAIDs should be avoided |
|  | **14** | The use of NSAIDs in the perioperative period for frail patients undergoing surgery for fractures is recommended within multimodal analgesia protocols to reduce opioid intake and limit the onset of complications, including delirium |
|  | **15** | Short-term administration (<10–15 days) of an NSAID in the postoperative period and upon discharge of a patient operated on for a fracture is not associated with a significant risk of pseudoarthrosis. |
| Domain 4 | **16** | The presence of cardiovascular risk factors or pathology in patients with chronic orthopedic pain requires a carefully personalized choice of NSAID. |
|  | **17** | Ketoprofen shows the best cardiovascular tolerability profile compared to other NSAIDs |
|  | **18** | The potential interference of NSAIDs with platelet aggregation processes requires particular attention in patients receiving ASA therapy |
|  | **19** | The presence of impaired renal function in patients with chronic orthopedic pain requires a carefully personalized choice of NSAID and the patient's hydration must be monitored. |
|  | **20** | The control of oncological pain in orthopedics may include the use of NSAIDs as part of a multimodal analgesic strategy |

**List of statements reaching agreement of the panel in first round**

|  | **Statement** | **Tertiles** | | |
| --- | --- | --- | --- | --- |
|  |  | **1-2 (%)** | **3-4 (%)** | **5-6 (%)** |
| **1** | NSAIDs act on the genesis of inflammatory pain, on spinal transmission, and activate descending inhibitory pathways, with class-specific effects | 0.0 | 18.5 | 81.5 |
| **4** | The duration of NSAID therapy for the treatment of orthopedic pain should be limited in time (cycles of a maximum of 7–10 days), and the treatment should be included within a multimodal approach | 0.0 | 11.1 | 88.9 |
| **5** | NSAID treatment for osteoarticular pain while awaiting surgery should be performed starting with the minimum effective doses and/or in repeated short cycles | 0.0 | 22.2 | 77.8 |
| **9** | In patients with severe osteoarthritis (Kellgren–Lawrence >3) awaiting surgery, therapy may include treatment with NSAIDs or paracetamol combined with instrumental and manual physiotherapy techniques. Referral to a pain therapist may be appropriate | 3.7 | 14.8 | 81.5 |
| **10** | Early functional recovery requires analgesic continuity with dose adjustment and, if needed, the addition of ‘rescue’ medications, including NSAIDs, considering ERAS protocols | 0.0 | 22.2 | 77.8 |
| **12** | In patients with severe osteoarthritis, unresponsive to physiotherapy treatments, intolerant to NSAIDs, the use of paracetamol and opioids is advisable, even in combination | 0.0 | 7.4 | 92.6 |
| **14** | The use of NSAIDs in the perioperative period for frail patients undergoing surgery for fractures is recommended within multimodal analgesia protocols to reduce opioid intake and limit the onset of complications, including delirium | 0.0 | 14.8 | 85.2 |
| **15** | Short-term administration (<10–15 days) of an NSAID in the postoperative period and upon discharge of a patient operated on for a fracture is not associated with a significant risk of pseudoarthrosis | 3.7 | 11.1 | 85.2 |
| **16** | The presence of cardiovascular risk factors or pathology in patients with chronic orthopedic pain requires a carefully personalized choice of NSAID | 0.0 | 7.4 | 92.6 |
| **18** | The potential interference of NSAIDs with platelet aggregation processes requires particular attention in patients receiving ASA therapy | 0.0 | 7.4 | 92.6 |
| **19** | The presence of impaired renal function in patients with chronic orthopedic pain requires a carefully personalized choice of NSAID and the patient's hydration must be monitored | 0.0 | 11.1 | 88.9 |
| **20** | The control of oncological pain in orthopedics may include the use of NSAIDs as part of a multimodal analgesic strategy | 0.0 | 3.7 | 96.3 |

**Results of the first round, first domain**

|  |  | | **Tertiles** | | |
| --- | --- | --- | --- | --- | --- |
|  | **Statement** | **Judgement** | **1-2 (%)** | **3-4 (%)** | **5-6 (%)** |
| **1** | NSAIDs act on the genesis of inflammatory pain, on spinal transmission, and activate descending inhibitory pathways, with class-specific effects | Agreement | 0.0 | 18.5 | 81.5 |
| **2** | The efficacy of NSAIDs in musculoskeletal and joint pain is comparable at equivalent doses | Disagreement | 0.0 | 25.9 | 74.1 |
| **3** | Gastrointestinal adverse events of NSAIDs are dose-dependent and drug-specific, regardless of the route of administration | Disagreement | 0.0 | 25.9 | 74.1 |
| **4** | The duration of NSAID therapy for the treatment of orthopedic pain should be limited in time (cycles of a maximum of 7–10 days), and the treatment should be included within a multimodal approach | Agreement | 0.0 | 11.1 | 88.9 |

**Results of the first round, second domain**

|  |  | | **Tertiles** | | |
| --- | --- | --- | --- | --- | --- |
|  | **Statement** | **Judgement** | **1-2 (%)** | **3-4 (%)** | **5-6(%)** |
| **5** | NSAID treatment for osteoarticular pain while awaiting surgery should be performed starting with the minimum effective doses and/or in repeated short cycles | Agreement | 0.0 | 22.2 | 77.8 |
| **6** | During intervals between therapy cycles, or in patients with contraindications to the use of NSAIDs, the use of SYSADOA may be beneficial | Disagreement | 3.7 | 51.9 | 44.4 |
| **7** | SYSADOA can be used in combination with NSAIDs as part of conservative pain-management strategies | Disagreement | 7.4 | 59.3 | 33.3 |
| **8** | In patients with moderate osteoarthritis (Kellgren–Lawrence up to grade 3) awaiting surgical intervention, multimodal therapy may include treatment with NSAIDs or paracetamol combined with intra-articular injection techniques using hyaluronic acid or PRP, provided they are performed at least three months before surgery | Disagreement | 14.8 | 29.6 | 55.6 |
| **9** | In patients with severe osteoarthritis (Kellgren–Lawrence >3) awaiting surgery, therapy may include treatment with NSAIDs or paracetamol combined with instrumental and manual physiotherapy techniques. Referral to a pain therapist may be appropriate | Agreement | 3.7 | 14.8 | 81.5 |
| **10** | Early functional recovery requires analgesic continuity with dose adjustment and, if needed, the addition of ‘rescue’ medications, including NSAIDs, considering ERAS protocols | Agreement | 0.0 | 22.2 | 77.8 |
| **11** | In the context of social reintegration, in cases of acute pain with NRS >4, the most suitable NSAID to use as needed is ketoprofen lysine salt, in terms of risk–benefit ratio | Disagreement | 0.0 | 44.4 | 55.6 |
| **12** | In patients with severe osteoarthritis, unresponsive to physiotherapy treatments, intolerant to NSAIDs, the use of paracetamol and opioids is advisable, even in combination | Agreement | 0.0 | 7.4 | 92.6 |

**Results of the first round, third domain**

|  |  | | **Tertiles** | | |
| --- | --- | --- | --- | --- | --- |
|  | **Statement** | **Judgement** | **1-2 (%)** | **3-4 (%)** | **5-6 (%)** |
| **13** | In patients at risk of developing stress fractures, chronic use of NSAIDs should be avoided | Disagreement | 0.0 | 33.3 | 66.7 |
| **14** | The use of NSAIDs in the perioperative period for frail patients undergoing surgery for fractures is recommended within multimodal analgesia protocols to reduce opioid intake and limit the onset of complications, including delirium | Agreement | 0.0 | 14.8 | 85.2 |
| **15** | Short-term administration (<10–15 days) of an NSAID in the postoperative period and upon discharge of a patient operated on for a fracture is not associated with a significant risk of pseudoarthrosis | Agreement | 3.7 | 11.1 | 85.2 |

**Results of the first round, fourth domain**

|  |  | | **Tertiles** | | |
| --- | --- | --- | --- | --- | --- |
|  | **Statement** | **Judgement** | **1-2 (%)** | **3-4 (%)** | **5-6 (%)** |
| **16** | The presence of cardiovascular risk factors or pathology in patients with chronic orthopedic pain requires a carefully personalized choice of NSAID | Agreement | 0.0 | 7.4 | 92.6 |
| **17** | Ketoprofen shows the best cardiovascular tolerability profile compared to other NSAIDs | Disagreement | 0.0 | 25.9 | 74.1 |
| **18** | The potential interference of NSAIDs with platelet aggregation processes requires particular attention in patients receiving ASA therapy | Agreement | 0.0 | 7.4 | 92.6 |
| **19** | The presence of impaired renal function in patients with chronic orthopedic pain requires a carefully personalized choice of NSAID and the patient's hydration must be monitored | Agreement | 0.0 | 11.1 | 88.9 |
| **20** | The control of oncological pain in orthopedics may include the use of NSAIDs as part of a multimodal analgesic strategy | Agreement | 0.0 | 3.7 | 96.3 |

**List of the statements modified after the first round, reaching agreement of the panel in the second round**

|  | **Statement** | **Tertiles** | | |
| --- | --- | --- | --- | --- |
|  |  | **1-2 (%)** | **3-4 (%)** | **5-6 (%)** |
| **2** | The efficacy of NSAIDs in treating musculoskeletal and joint pain is similar when equivalent doses are used. The equivalent dose is the amount of drug that produces a similar analgesic and anti-inflammatory effect compared to another NSAID | 8.7 | 4.3 | 87.0 |
| **3** | The gastrointestinal adverse events of systemic NSAIDs are dose-dependent and drug-specific for every route of administration except the topical one | 0.0 | 4.3 | 95.7 |
| **7** | SYSADOAs when administered as part of conservative treatment strategies for osteoarthritic pain, can help reduce the use of NSAIDs | 4.3 | 4.3 | 91.3 |
| **11** | At discharge after surgery, in the management of pain, ketoprofen lysine salt represents the NSAID with the best risk-benefit ratio especially in treatments lasting up to 7–14 days | 0.0 | 0.0 | 100.0 |
| **13** | In patients at risk of developing stress fractures, regular use of NSAIDs should be limited | 0.0 | 0.0 | 100.0 |
| **17** | Ketoprofen has a lower cardiovascular risk than other NSAIDs | 0.0 | 4.3 | 95.7 |
